# Complete Genome Sequence of Pathogenic Guinea Pig Cytomegalovirus from Salivary Gland Homogenates of Infected Animals

**DOI:** 10.1128/genomeA.00054-13

**Published:** 2013-03-14

**Authors:** Dongmei Yang, Kristen Tamburro, Dirk Dittmer, Xiaohong Cui, Michael A. McVoy, Nelmary Hernandez-Alvarado, Mark R. Schleiss

**Affiliations:** a University of North Carolina School of Medicine, Department of Microbiology and Immunology, Chapel Hill, North Carolina, USA; b Virginia Commonwealth University School of Medicine, Department of Pediatrics, Richmond, Virginia, USA; c University of Minnesota Medical School, Department of Pediatrics, Division of Pediatric Infectious Diseases and Immunology, Minneapolis, Minnesota, USA

## Abstract

The sequence of guinea pig cytomegalovirus (GPCMV) was determined by direct sequencing of salivary gland homogenates obtained following sustained, serial *in vivo* passage of pathogenic virus in guinea pigs. The 233,501-nucleotide salivary gland (SG) genome was noted to have 11 differences compared to the tissue culture-passaged virus, although no variations were noted in putative protein coding sequences.

## GENOME ANNOUNCEMENT

Guinea pig cytomegalovirus (GPCMV) is a model for congenital CMV infection, an important cause of disability in newborns (1). Strain 22122, isolated by Hartley in 1957 (ATCC, VR-682), is the only characterized GPCMV strain (2). Salivary gland (SG) extracts are pathogenic, while tissue culture (TC)-passaged viruses are significantly attenuated. Existing genomic sequences were derived from TC-adapted GPCMV (3) or ATCC virus (AB592928), which was also passaged multiple times in cell culture prior to submission to ATCC (4). Therefore, we sought to determine the sequence of pathogenic GPCMV, obtained originally from the ATCC strain 21222 stock but maintained exclusively by serial passage in animals. Sequencing was performed directly on DNA purified from the SG homogenate, with no intervening passage in TC.

Approximately 35 serial SG passages were made in animals over a 25-year period (1985 to 2010) in Cincinnati, OH, and Minneapolis, MN, in strain 2 guinea pigs. SG tissue was minced, homogenized, clarified by centrifugation, and stored at −80°C. 350 PFU of this SG stock has been shown to produce efficient fetal infection and disease (5). Virions in the SG homogenate were pelleted by ultracentrifugation; treated with DNase, proteinase K, and RNase; phenol/chloroform extracted; alcohol precipitated; and sequenced on an Illumina Genome Analyzer. 119,721,518 raw reads were obtained; reads under 70 bp were discarded. Of the remaining 83,249,482 reads, 30,674,158 matched AB592928, which was used as a template to assemble the full-length SG sequence. Average coverage was 9,609.

Initial comparison of the SG sequence to AB592928 suggested deletions within a ∼1.2-kb region of complex repeats thought to comprise the GPCMV origin of replication. Given the short lengths of the Illumina reads, these apparent deletions likely represented artifacts of sequence assembly, as within repeated sequences the assembly software could not orient overlapping reads to properly reconstruct the region. To address this concern, SG homogenate was PCR amplified using primers flanking the repeat region (5′-TGG GTG TGG GAG TGG CTT TG-3′ and 5′-TCG GTC TGG ATG CGT GTT G-3′) with Vent polymerase, and multiple independently isolated clones were sequenced. The size of the PCR product (∼1.2 kb) was consistent with that predicted from AB582828 and, following TA cloning and Sanger sequencing, this sequence was incorporated into the final SG sequence.

Comparison of the AB592928 with SG-22122 revealed eleven differences: four nucleotide substitutions, one clarification of an ambiguous base, one nucleotide insertion, and five 1- to 2-nucleotide differences in poly(T) tracts. None of the differences altered codons in currently annotated open reading frames.

We annotated an alternative start codon for *gp148*, a major histocompatibility complex (MHC) I homolog. This removed a cluster of sixteen 7-bp repeats near the 5′ end of the previous annotation, resulting in a shorter protein of 350  amino acids (aa). Other putative open reading frames (ORFs) are similar between the SG-22122 and AB592928 sequences, with the exception that analysis of SG-22122 revealed two additional previously unannotated gp138 family ORFs (gp138.1, gp138.3). All three members of this gp138 ORF cluster were noted to bear predicted structural similarity to immunoglobulin variable (IgV) domains from the Ig superfamily of proteins, suggesting a potential role for these gene products in immune evasion/immune modulation.

In summary, the sequence of the pathogenic SG genome reveals surprisingly few differences from ATCC stock. A similar level of stability has been noted upon whole-genome sequencing for murine CMV (6). For GPCMV, attenuation following tissue culture passage may be related to deletions in key pathogenesis genes (7). Additional studies are required to elucidate the molecular basis of GPCMV attenuation following TC adaptation.

### Nucleotide sequence accession number.

The genome sequence for this strain has been deposited with GenBank under the accession number KC503762.
